# Crosslinked Carboxymethyl Sago Starch/Citric Acid Hydrogel for Sorption of Pb^2+^, Cu^2+^, Ni^2+^ and Zn^2+^ from Aqueous Solution

**DOI:** 10.3390/polym12112465

**Published:** 2020-10-24

**Authors:** Amyrah Auni Keirudin, Norhazlin Zainuddin, Nor Azah Yusof

**Affiliations:** Chemistry Department, Faculty of Science, Universiti Putra Malaysia, Serdang 43400 UPM, Selangor, Malaysia; amyrahauni@gmail.com (A.A.K.); azahy@upm.edu.my (N.A.Y.)

**Keywords:** carboxymethyl starch, sago, citric acid, hydrogel, heavy metal, sorption

## Abstract

In the present study, CMSS (carboxymethyl sago starch)-based hydrogel was synthesized by crosslinking with citric acid via esterification and then applied as a metal sorbent to overcome excessive heavy metal pollution. The CMSS/CA (carboxymethyl sago starch/citric acid) hydrogel was characterized by Fourier Transform Infrared (FT-IR), scanning electron microscopy (SEM), thermogravimetric analysis (TGA) and X-ray diffraction (XRD). The absorption band at 1726 cm^−1^ was observed in the FT-IR spectrum of CMSS/CA hydrogel and indicated ester bonds formed. Further findings show that the cross-linkages in the CMSS/CA hydrogel increased the thermal stability of CMSS and various sizes of pores were also shown in the SEM micrograph. Conversely, the removal of heavy metals was analyzed using Inductively Coupled Plasma-Optic Emission Spectra (ICP-OES). The effects of the pH of the metal solution, contact time, initial concentration of the metal ions and temperature on the sorption capacity were investigated. Under optimum condition, the sorption capacity of Pb^2+^, Cu^2+^, Ni^2+^ and Zn^2+^ onto CMSS/CA hydrogel were 64.48, 36.56, 16.21, 18.45 mg/g, respectively. The experiments demonstrated that CMSS/CA hydrogel has high selectivity towards Pb^2+^ in both non-competitive and competitive conditions. In conclusion, the CMSS/CA hydrogel as a natural based heavy metal sorption material exhibited a promising performance, especially in the sorption of Pb^2+^ for wastewater treatment.

## 1. Introduction

Developing countries like Malaysia have been exposed to heavy metal pollution that comes from different sources, like agriculture, industrial and domestic activities, which predominantly contribute to a high concentration of heavy metal ions discharge through sewage and straight into water source [1,2,3]. Accumulation of these hazardous inorganic pollutants could eventually enter the food chains and cause a detrimental effect on living organisms and human health with high toxicity and carcinogenicity [4]. Through strict legislation controlling the heavy metal concentration before discharge into nature, many conventional wastewater treatments have been enacted, such as chemical precipitation, ion exchange, adsorption on activated carbon, solvent extraction, and membrane filtration [5,6]. However, these treatments consume high energy and cost operation, incomplete removal, especially in low concentration, as well as produce large quantities of toxic sludge that unintentionally become second pollution [7,8]. 

In removing the heavy metal from inorganic effluent, sorption becomes one of effective alternative techniques. The sorption process can be described as a physical or chemical interaction that occurs when the mass transfers a substance from the liquid phase to the surface of a solid. Conversely, the sorbent can be derived from a low-cost material, such as agricultural waste, natural material, modified biopolymer and industrial by-product [9,10]. As a result, there are rising numbers of studies on the sorption of heavy metal by polysaccharides-based hydrogel owing to their accessibility, economical, renewability, biodegradability and environmental compatibility to practice green chemistry [3,11,12].

Hydrogel, which is a hydrophilic gel possesses a three-dimensional polymeric network capable of absorbing and retaining a significant amount of water or aqueous solution. It is composed of a physically or chemically crosslinked polymer network. The term ‘network’ in hydrogels points out crosslinks that must exist in order to prevent the dissolution of polymeric chains [13]. However, previous studies have shown various type of polysaccharides-based hydrogel that has been used as a heavy metal sorbent, for example, gellan gum [3], potato starch [14,15], gum tragacanth [9] and cellulose [16]. The key feature to facilitate high sorption capacity is the presence of hydrophilic functional groups, like amine, hydroxyl, carboxylic acid or sulfonic acid acting as chelating agent for removal of heavy metal ions from aqueous solution [17].

Native sago starch, isolated from sago palm, draws innumerable attention as a promising natural polymer. As such, this polysaccharide was chosen as it is one of the rich sources for low-cost sorbent that also biodegradable. In general, native sago starch has limited utilization in heavy metal sorption unless the physio-chemical and physico-chemical properties are modified through several techniques. The availability of three hydroxyl groups at position C2, C3 and C6 in starch backbone become the targeting sites for the alteration of starch structure. Additionally, Carboxymethyl Sago Starch (CMSS) is one of the sago starch derivatives with a carboxymethyl functional group on the starch backbones [18]. CMSS have been constantly used as a natural polymer in hydrogel synthesis due to the advantages of low rates of retrogradation, increase thermal stability and hydrophilic functional group [19,20]. However, despite the favourable key structure of carboxylic COO^−^ groups that can be bind to divalent metal ions, yet CMSS has low mechanical strength and instability in the gel form [21]. A chemically crosslinked hydrogel is crucial for forming a permanent gel with high porosity which plays prominent roles to enhance pollutant sorption [22]. 

Naturally, harmless crosslinker, such as citric acid preferably used rather than intrinsic toxic of common crosslinkers, such as epichlorohydrin, phosphorous chloride or formaldehyde that may give potential risks to the environment [23,24]. The significant benefit of choosing citric acid as crosslinking agent is because it has a good affinity towards metal ions [25]. Furthermore, it composed of carboxyl groups that esterified into cyclic anhydride, as well as the active sites for crosslinking reaction with hydroxyl groups in CMSS [26]. More so, the solubility of citric acid crosslinked starch declined as the hydroxyl group decreased from the breakage in glycosidic linkage, thus the introduction of the new bulky functional group resulted in retarding the mobility of starch [27].

In the present study, CMSS/CA hydrogel was synthesized by crosslinking the CMSS with citric acid and then applied it as a metal sorbent to study its sorption properties in removing Pb^2+^, Cu^2+^, Ni^2+^ and Zn^2+^ from aqueous solution. Nevertheless, sago starch and citric acid are considered to be excellent materials to be used in the application of heavy metal removal because of their biodegradable as well as renewable compared to a synthetic polymer which may create secondary pollution. As such, the usage of low-cost and bio-sorbent like CMSS/CA hydrogel is crucial for industries as it brings benefits in terms of operation cost and environmentally friendly.

Furthermore, the properties of CMSS/CA hydrogel were characterized by Fourier-transform infrared (FT-IR), scanning electron microscopy (SEM), thermogravimetric analysis (TGA) and X-ray diffraction (XRD). The sorption capacity of CMSS/CA hydrogel was evaluated through several parameters; pH of the metal solution, contact time, initial metal ion concentration and temperature. Also, the sorption behavior of CMSS/CA hydrogel was discussed with kinetics, isotherm and thermodynamics studies. Moreover, the selectivity of sorbent towards different heavy metal ions and desorption of heavy metal ion were also studied.

## 2. Materials and Methods

### 2.1. Materials 

A food-grade sago starch powder was purchased from C.L Nee Sago Industries Sdn. Bhd., Malaysia. Solvents, such as isopropanol, methanol, absolute ethanol and acids like glacial acetic acid, nitric acid, hydrochloric acid, monohydrate citric acid and sodium hydroxide were acquired from R&M Chemicals (Essex, UK). Other than this, phenolphthalein and diphenylamine reagent were also obtained from Friendemann Schimidt (Parkwood, WA, USA), buffer solution from Systerm ReadilPur (Malaysia) and sodium monochloroacetate from Sigma-Aldrich Co. (St. Louis, MO, USA) respectively. Furthermore, metal standard solutions of lead, copper, nickel and zinc were supplied by Fisher Chemicals (Loughborough, UK). All chemicals used in this experiment were analytical grade and used without further purification. Deionized and distilled water were used throughout the experiment.

### 2.2. Preparation of CMSS

The modification of sago starch by carboxymethylation method was done according to the procedure of Basri et al. [28]. CMSS was synthesized via the etherification reaction of an active hydroxyl group in starch with sodium monochloroacetate (SMCA), which resulted in the formation of ether link (R–OR). This procedure is commonly done in an alkaline environment using sodium hydroxide as a chemical substitution initiator or catalyzing agent. Based on Williamson ether synthesis, and the carboxymethylation was derived from native starch proceed by two main step reactions proposed by Lviii et al. [29]: (i) alkalization of starch by sodium hydroxide, NaOH and (ii) etherification of starch by SMCA. The CMSS was washed three times with methanol. From the measurement of the average number sodium carboxymethyl group substituted per anhydroglucose unit (AGU), the degree of substitution (DS) of CMSS prepared in the present study was 0.82. 

### 2.3. Synthesis of CMSS/CA Hydrogel

**Figure 1:**
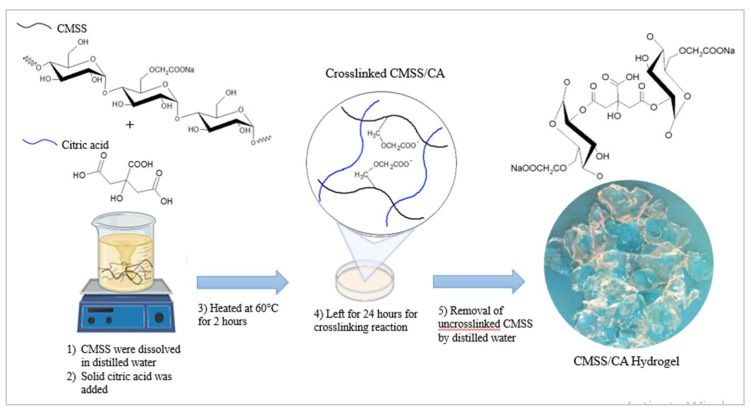
Schematic diagram describing the preparation steps for the synthesis of CMSS/CA hydrogel.

For the synthesis of CMSS/CA hydrogel, initially, 60% (w/v) of CMSS (degree of substitution of 0.82) were dissolved in distilled water by stirring at room temperature until a clear and viscous solution was obtained. Thereafter, 10% (w/w) of solid citric acid was added into the solution and stirred. The mixture was left for 1 h so that it can settle down, and then heated gently at 60 °C, while stirring for another 2 h. Afterwards, the mixture was poured into the petri dish and left for 48 h at 50 °C for crosslinking reaction to continue. The uncrosslinked CMSS and excess citric acid were then removed by washing with distilled water. Finally, the pure CMSS/CA hydrogel was dried in the oven at 60 °C. Figure 1 shows the preparation steps for the synthesis of CMSS/CA hydrogel.

### 2.4. Characterization of CMSS/CA Hydrogel

The functional groups of sago starch, CMSS and CMSS/CA hydrogel can be characterized by using FTIR spectrometer (100 Series PerkinElmer, Waltham, MA, USA). For the present study, the data was collected in the spectral range of 4000–280 cm^−1^ using the Universal Attenuated Total Reflectance (UTAR, PerkinElmer, Waltham, MA, USA ) sampling technique. The identification of crystallinity and characterization in each sample of sago starch, CMSS and CMSS/CA hydrogel based on diffraction pattern was equally examined by using an X-ray Diffractometer. The sample was exposed to Cu Kα (λ = 1.5418 Å) radiation at room temperature operated at 30 kV and 30 mA within scanning range of 2° to 30° with the rate of 2°/min in continuous scan mode. Meanwhile, the distinctive surface morphology of sago starch, CMSS and CMSS/CA hydrogel samples were identified via FEI, Quanta 400 scanning electron microscopy. Accordingly, the swollen hydrogel was treated by freeze-drying to remove out the water in the sample before coated with gold for the samples to become conductive to be analyzed at a magnification between 100–1000 ×. The decomposition and thermal stability of sago starch, CMSS as well as CMSS/CA hydrogel were quantitatively examined by using Mettler Toledo TGA/SDTA-851^e^/STAR^e^. The sample was heated from 50 °C up to 800 °C at a rate of 10 °C/min in the flow of inert nitrogen. 

### 2.5. Sorption Procedure 

In addition, the sorption of heavy metal ions; Pb^2+^, Cu^2+^, Ni^2+^ and Zn^2+^ from aqueous solution onto CMSS/CA hydrogel was performed using the batch equilibrium method, in a 50 mL centrifuge, 0.05 g of CMSS/CA hydrogel which was placed with 25 mL of heavy metal ion solution at initial concentration of 20 ppm and pH 1. The pH was adjusted with 1.0 M NaOH or HCl, while the centrifuge tube was then agitated in a water bath shaker at a constant rate (160 rpm) at 27 °C for 15 min, allowing the CMSS/CA hydrogel-metal ions complex to form. Afterwards, the supernatant was separated from the hydrogel-metal complex by filtering with filter paper. The residual heavy metal concentration in the filtrate was analyzed by Inductively Coupled Plasma-Optical Emission Spectrometer (ICP-OES) (Perkin-Elmer Optima 2100 DV, PerkinElmer, Waltham, MA, USA). The experiments were done in triplicate and data were recorded as a mean. At the end, the removal percentage of metal ions and sorption capacity at equilibrium were calculated [30],

(1) $$\%\;\mathrm{Removal}\;=\;\frac{C_{o\;}{-C}_{e}}{C_{o}}\times 100$$

(2) $$q_{e}=\frac{{(C}_{o}{-C}_{e\;})}{m}\times V$$

where q_e_ (mg/g) is the amount of metal ions sorbed per unit mass of the sorbent, C_o_ and C_e_ (mg/L) are the initial and final or equilibrium concentration of metal ions in solution respectively, V (L) is the volume of metal ions solution and m (g) is the mass of sorbent. 

Meanwhile, metal ion sorption was studied as a function of pH of metal ions solution (pH 1 to pH 5), reaction time (15 to 75 min), initial metal ion concentration (20 to 150 ppm) and reaction temperature (27 to 55 °C).

### 2.6. Selectivity Study

The selectivity study was done by determining the removal percentage in single metal ions solution and multivalent metal ions solution containing a mixture of Pb^2+^, Cu^2+^, Ni^2+^ and Zn^2+^. Under the non-competitive condition, 0.05 g of sorbent which was added into 25 mL of single metal ions solution with pH 4 and 20 ppm, thereafter agitated in a water bath shaker at 160 rpm and room temperature for 30 min. While under competitive condition, mixed metal ions solution was used with every single metal ion concentration of 20 ppm. The same experimental conditions were applied for a fair comparison with the competitive condition. The CMSS/CA-metal ions complex was filtered, and the final concentration of the filtrate was determined by ICP-OES.

### 2.7. Desorption Study

The desorption of heavy metal ions was carried out in a 0.1 M HCl solution and functions as a desorption medium. The CMSS/CA hydrogel loaded heavy metal was collected and agitated in desorption medium at 600 rpm at room temperature for 30 min, according to the method by Niu et al. [31]. The final metal ion concentration in the desorption medium was analyzed by ICP-OES and the percentage of metal ions desorption was calculated by the following equation,

(3) $$\%\mathrm{Desorption}=\;\frac{C_{\mathrm{des}}}{C_{\mathrm{ad}}}\times 100\%$$

where C_des_ (ppm) is the amount of metal ions desorb and C_ad_ (ppm) is the amount of metal ion adsorbed.

## 3. Results and Discussion 

### 3.1. Characterization of CMSS/CA Hydrogel

#### 3.1.1. FTIR Analysis

**Figure 2:**
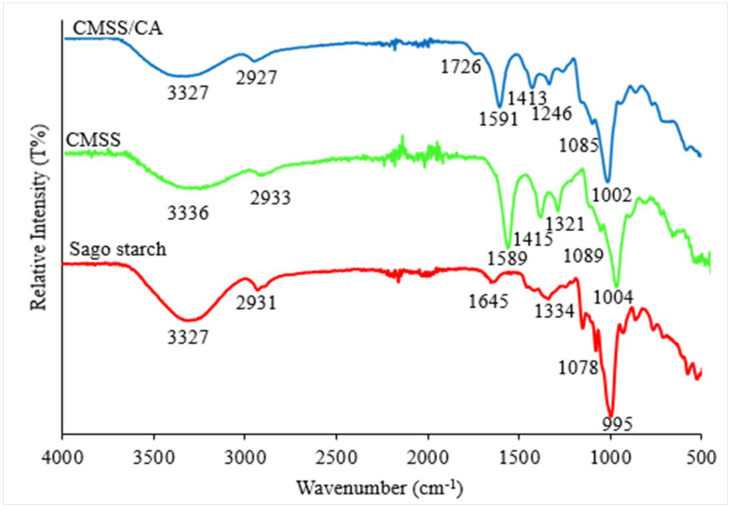
FTIR spectra of sago starch, CMSS and CMSS/CA hydrogel.

FTIR spectra of sago starch, CMSS and CMSS/CA hydrogel are depicted in Figure 2. The FTIR spectrum of sago starch shows a broad absorption band at 3327 cm^−1^_,_ indicating the O–H stretching. Generally, the O–H absorption band was at the range of 3700–3500 cm^−1^ but as mentioned by Zainuddin [32], the band shifted to a lower frequency of 3400–3200 cm^−1^. This might be due to the O–H bond that became weaken by intermolecular hydrogen bonds in the glycosidic ring. The C–H stretching band at 2931 cm^−1^ and weak absorption band at 1645 cm^−1^ indicated the presence of tightly bound water in the starch molecule. While, the absorption band at 1334 cm^−1^ was attributed to the –CH_2_ symmetry. Broad absorption band in the range of 1100–990 cm^−1^ indicated the C–O stretching in C–O–C and C–O–H in glycosidic rings.

The FTIR spectrum of CMSS has all typical absorption bands of sago starch with additional bands resulting from the carboxymethylation reaction. The O–H stretching in CMSS at 3336 cm^−1^ reduced the intensity due to the substitution of the O–H group with –CH_2_COONa during etherification. A new absorption band at 1589 cm^−1^ represented the CH_2_COONa on the starch backbone. The IR wavenumber of the carbonyl group was lower than the value of parent carboxylic owing to the resonance effect which is formed from the carboxylate group [28]. The IR spectrum of CMSS/CA hydrogel showed a new absorption band at 1726 cm^−1^ and indicated the overlapped C=O stretching of free carboxylic acid as well as ester group [26]. The intensity of COO^−^ group at band 1591 cm^−1^ that was reduced might be due to the –COONa in CMSS converted to COOH. Last, during the crosslinking reaction, the replacement of Na with hydrogen from acid was also reported by Basri et al. [28]. A new absorption band at 1246 cm^−1^ denoted the C–O stretching for the ester group.

#### 3.1.2. XRD Analysis 

**Figure 3:**
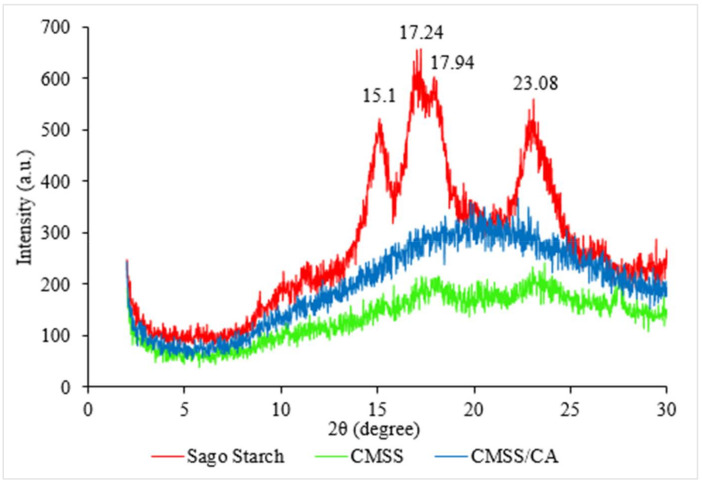
Diffraction pattern of sago starch, CMSS and CMSS/CA hydrogel.

Figure 3 shows the X-ray diffraction (XRD) analysis of the diffraction pattern of amorphous or crystallinity phase for sago starch, CMSS and CMSS/CA hydrogel. The native sago starch diffractogram depicts characteristics of the C-type crystallinity pattern, and it is a combination of A- and B-type pattern. Nevertheless, strong reflection intensity can be observed at 2θ value of 15.1°, 17.2°, 17.9° and 23.1° where the characteristic peaks of native sago starch in the semi-crystalline phase. This result is in good agreement with the previous studies by Othman et al. [33] and Uthumporn et al. [34]. The diffraction pattern of CMSS shows broaden and reduction in intensities of crystalline peaks from native starch. It is attributed to the destruction of the crystalline structure of the original sago and transformed into an amorphous phase. According to Liu et al. [35], the substitution of carboxymethyl into hydroxyl groups breaks the H–bond and greatly reduced the crystallinity. The reduction in the degree of crystallinity was due to carboxymethylation as reported for potato starch by Zhang et al. [24]. While, for the CMSS/CA hydrogel, the XRD pattern shows a broad peak distribution and no sharp peak that indicates the amorphous phase of CMSS, thus, the increase in the intensity can be attributed to the presence of the citric acid as a crosslinker in the network of the CMSS. However, the amorphous region of CMSS/CA hydrogel is crucial for water to become more accessible into the network of the hydrogel. While, the loss of crystallinity allows the diffusion of water into the hydrogel and swell. 

#### 3.1.3. SEM Analysis

**Figure 4:**
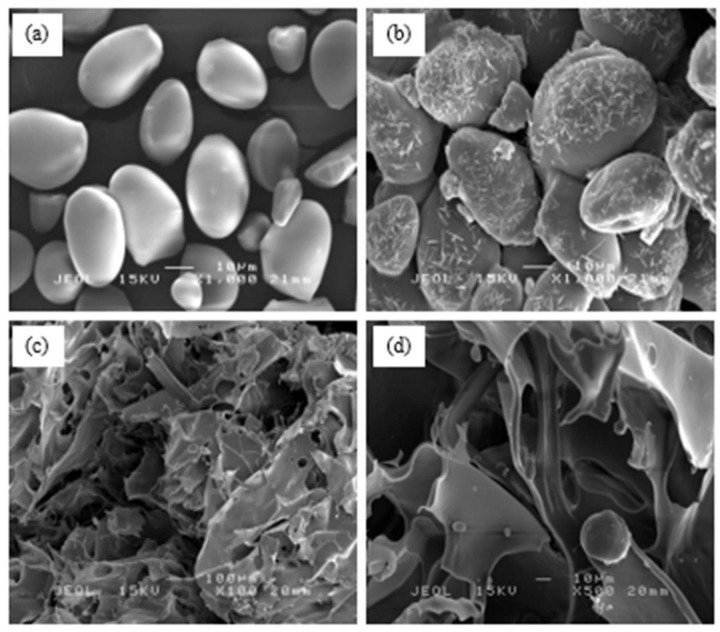
SEM micrograph of (**a**) sago starch at 1000x magnification; (**b**) CMSS at 1000 × magnification; (**c**) CMSS/CA hydrogel at 100 × magnification and (**d**) CMSS/CA hydrogel at 500 × magnification.

Scanning electron microscopy (SEM) focuses on observing the surface morphology of the sample. For sago starch (Figure 4a), the fine granule has a smooth surface and mainly ovoid with a truncated side. A similar micrograph of native starch can be seen from previous studies by Othman et al. [33] and Uthumporn et al. [34]. Nevertheless, the appearance of CMSS (Figure 4b) was distorted and became agglomerate in an irregular shape. The surface of CMSS was relatively rough with fine fissures and acicular shape covered on the surface. This is probably due to the high degree of substitution of carboxymethyl group into the starch molecule. During carboxymethylation, sago starch was exposed to a strong alkali environment. Therefore, it significantly affected the morphology and granular disintegration, causing the destruction of crystallinity as shown in the XRD pattern. This finding is in good agreement with the morphology of carboxymethyl sago starch studied by Zainal et al. [14] and carboxymethyl chinese yam starch reported by Yanli et al. [36]. Besides, CMSS/CA hydrogels in Figure 4c,d have polymeric networks of hydrogel with an abundance of pores proved the formation of crosslinkages between citric acid and CMSS during esterification, and resulted in a more ester bonds in the hydrogel [37].

#### 3.1.4. TGA Analysis

**Figure 5:**
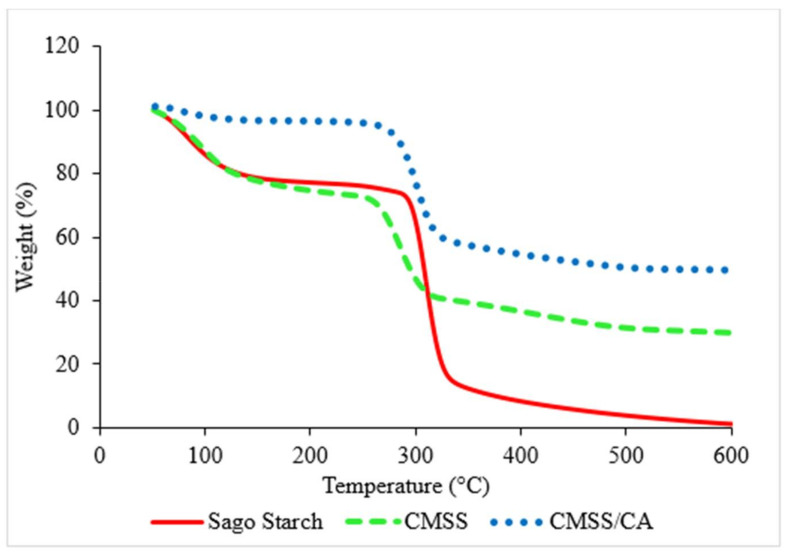
TGA thermogram of sago starch, CMSS and CMSS/CA hydrogel.

TGA thermograms of sago starch, CMSS and CMSS/CA hydrogel are depicted in Figure 5 to shows quantitative measurement on the weight change of sample against temperature. All TGA curves showed two major stages of decomposition. The first stage of decomposition occurred around 150.00 °C to 180.00 °C correlated to evaporation of water attached to the surface and entrapped water in the sample. The second stage took place around 178.00 °C to 550.00 °C, which corresponded to the degradation of the starch backbone.

**Figure 6:**
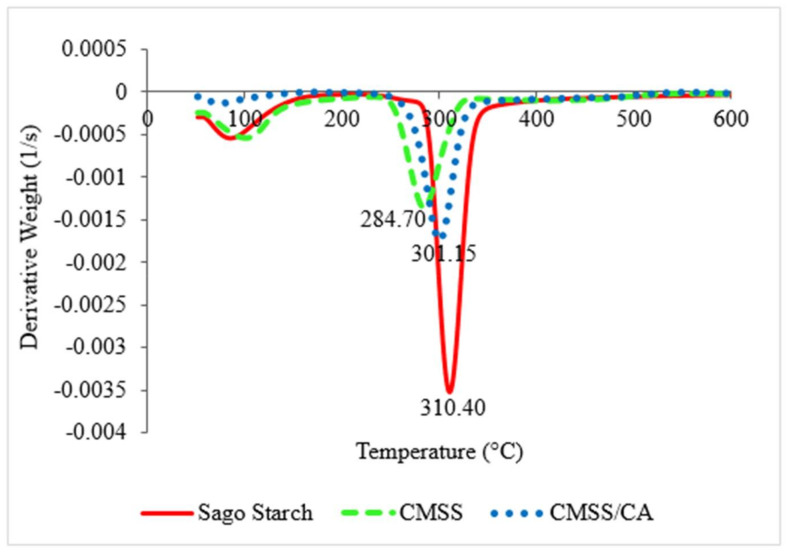
DTG thermogram of sago starch, CMSS and CMSS/CA hydrogel.

In Figure 6, the DTG thermogram presented the maximum decomposition for sago starch at 310.40 °C with a percentage weight loss of 67.26%. However, about 9.12% of residue remained in the sago starch was mainly composed of ash. For CMSS, the highest weight loss took place at 284.70 °C with 33.41% of total decomposition which is due to decarboxylation of CO_2_ from carboxymethyl in CMSS [38]. Also, the total of residue left from CMSS was about 21.13%, which is higher than sago starch owed to the additional substituted group (carboxymethyl and sodium salt) in CMSS. In contrast to sago starch, the maximum decomposition temperature of CMSS shifted to a lower temperature. As such, the conversion of sago starch into CMSS caused the amendment in the thermal behavior of CMSS. Similar results have been reported by Basri et al. [28] and Jamingan et al. [39], which stated that modified sago starch tends to have lower degradation temperature than native sago starch. Moreover, the replacement of CH_2_COONa^+^ into the hydroxyl group of sago starch distorted the crystalline region in a hydrogen bond [38]. That means the energy bonding of sago starch weakens in consequence of a reduction in the stability of CMSS to heat. Therefore, thermal stability and decomposition of CMSS decreased, compared to the sago starch. Conversely, the CMSS/CA hydrogel has higher thermal stability than CMSS as the maximum decomposition temperature of CMSS/CA hydrogel that is 301.15 °C compared to 284.70 °C for CMSS. Nevertheless, the higher temperature is required to decompose the CMSS/CA hydrogel in order to break the bonding of crosslinkages, which comprises ester and hydrogen bonding. While, the total remaining residue was 38.16% as a result of the high density of crosslink in the network of hydrogel [40] and the slow decomposition of citric acid crosslinked hydrogel [26].

### 3.2. Metal Ions Sorption by CMSS/CA Hydrogel

In the present research, the performance of heavy metal sorption was analyzed using the CMSS/CA hydrogel as the sorbent. The modification of sago starch into anionic starch, CMSS resulted in the addition of carboxylic COO^−^ groups that facilitate the active site for heavy metal binding. Moreover, the crosslinkages in the CMSS/CA hydrogel increase stability, and cause the presence of various sizes of pores that play a crucial role in sorption of heavy metal into the hydrogel.

Furthermore, the sorption procedure was studied by the batch equilibrium method using single metal ions of interest. The effect of sorption parameters including pH of metal ions solution, contact time, initial concentration metal ions and reaction temperature were investigated. Other conditions that were kept constant throughout the sorption procedure were 25 mL of metal ions solution, 0.05 g of the hydrogel and 160 rpm of rotation speed.

#### 3.2.1. Effect of pH

**Figure 7:**
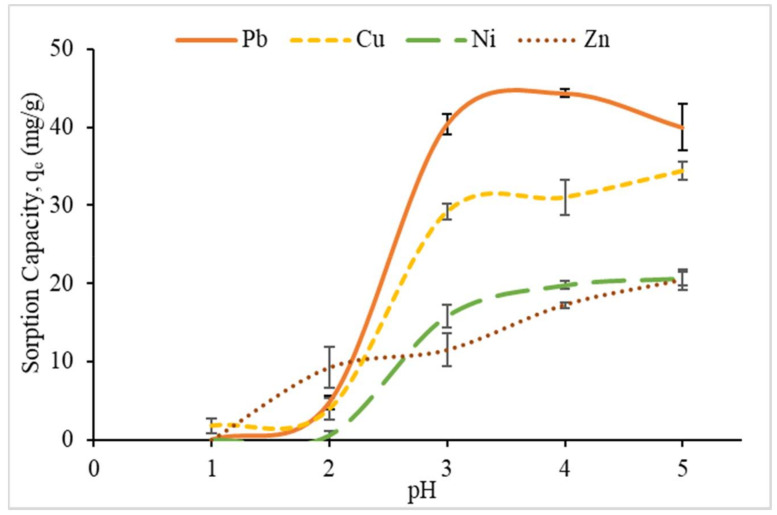
The effect of initial pH of metal ion solution on sorption capacity (q_e_).

The effect of pH metal ion solution on the sorption capacity of CMSS/CA (Figure 7) was investigated within the pH range from 1 to 5 with a constant amount of CMSS/CA hydrogel (0.05 g), contact time (30 min) and initial concentration of metal ions (100 ppm) at room temperature. The procedure was conducted only up to pH 5. This is because at pH higher than 5.5, excess hydroxide ions reacted with heavy metal ions and formed metal hydroxide precipitate. This will deflect the purpose of employing the sorption process and retarding the heavy metal removal [16].

**Figure 8:**
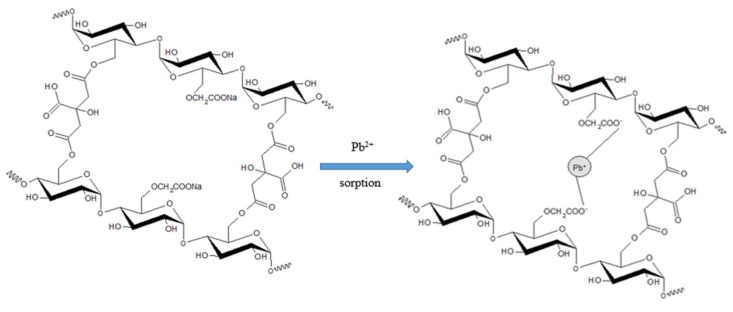
Proposed mechanism of ionic interaction between the active site of CMSS/CA hydrogel with metal ions.

In the present study, the sorption of heavy metal ions depends on the nature of charges of hydrogel since it involved ionic interaction between the negative charge from the carboxylate group of –COONa in hydrogel and the positive charge of divalent metal ions [15]. Figure 8 describes the proposed mechanism of ionic interaction for the sorption of metal ions on the active sites of the CMSS/CA hydrogel. At pH 1, the sorption capacity of CMSS/CA hydrogel on Pb^2+^, Ni^2+^ and Zn^2+^ was zero, while the sorption capacity of Cu^2+^ was only 1.8 mg/g. At low pH, the concentration of H^+^ was high and led to active sites of –COONa in carboxymethyl protonated into COOH and generated competition between H^+^ and metal ions for the sorption sites [9,41]. Therefore, the reduction of ligands for metal ions binding caused inhibition in metal ion sorption by the hydrogel [28]. Therefore, the sorption capacities of the CMSS/CA hydrogel at pH 1 and 2 were low. As the pH increased from pH 2 to 5, all the heavy metals showed a similar trend, where the sorption capacity gradually increased. At this stage, the concentration of hydrogen ions was low and led to carboxyl groups deprotonated into carboxylate ion (COO^−^). Hence, the ligand sites were available and the heavy metal ions no longer required to compete with H^+^ ions to form bonds with carboxylate ions in hydrogel [28]. Nevertheless, the optimum pH selected for next parameter was pH 4 for Pb^2+^, Cu^2+^ and Ni^2+^ while Zn^2+^ was pH 5 with sorption capacities of 44.37, 31.03, 19.81, and 20.42 mg/g, respectively.

#### 3.2.2. Effect of Contact Time and Kinetic Study on Metal Sorption

**Figure 9:**
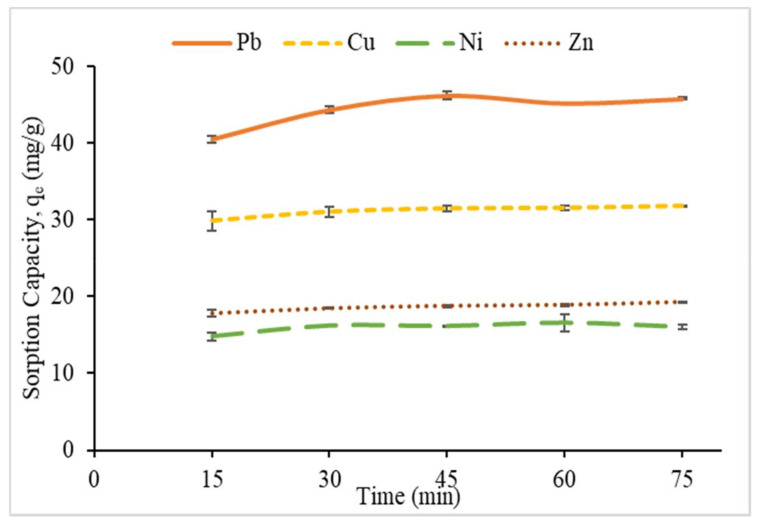
The effect of contact time on removal percentage.

The effect of contact time on the sorption capacity was carried out by varying contact time from 15 to 75 min in optimum pH and 100 ppm of metal ion concentration at room temperature. The results showed that the sorption capacity for all heavy metal ions increased for the first 45 min of contact time (Figure 9). El-Hamshary et al. [42] showed a similar result in their study and discussed that this behavior was due to a high number of free sorption sites at the surface of the sorbent in the first stage. This is because as the time prolonged, the sorption of Pb^2+^ and Ni^2+^ onto CMSS/CA hydrogel slightly decreased after 45 min, and 1 h, respectively. Sahraei et al. [9] explained that the decrease in sorption is probably because of the occupancy of active sites on sorbent and reached the equilibrium value. While, the sorption of Cu^2+^ and Zn^2+^ reached a plateau in value as the contact time elongated from 45 min and reaches its equilibrium at 75 min. The maximum sorption capacity of Pb^2+^ was 46.23 mg/g, Cu^2+^ was 31.79 mg/g, Ni^2+^ was 16.58 mg/g and Zn^2+^ was 19.27 mg/g.

In order to elucidate the kinetic of the sorption of heavy metal ions on CMSS/CA hydrogel, pseudo-first and pseudo-second order models were used. This is represented in an integrated form of rate equation 4, and 5, respectively as follows [30],

(4) $$\ln\left(q_{e}{-q}_{t}\right){=\mathrm{lnq}}_{e}{-k}_{1}t$$

(5) $$\frac{t}{q_{t}}=\frac{1}{k_{2}\;q_{e}^{2}}+\frac{t}{q_{e}}$$

where q_e_ and q_t_ (mg/g) are sorption capacity of the sorbent at equilibrium and at any time, while t (min), k_1_ (min^−1^) is the pseudo-first order rate constant and k_2_ (g mg^−1^ min^−1^) is the pseudo-second order rate constant. From the slope and intercept in the graph of pseudo-first order ln (q_e_−q_t_) against time and pseudo-second order t/q_t_ against time plotted, the rate constant of k_1_ and k_2_ were calculated shown in Table 1. For removal of Pb^2+^, Cu^2+^, Ni^2+^ and Zn^2+^ ions by CMSS/CA hydrogel, it reveals that the theoretical sorption capacity, and q_e,calc_ value calculated in the pseudo-second order are well in agreement with experimental data compared to the first-order model. For example, the experimental value of q_e_ for sorption of Pb^2+^ was 46.23 mg/g near to q_e_ calculation value for pseudo-second order which was 47.17 mg/g compared to pseudo-first order with q_e_ value of 7.737 mg/g. In addition, the correlation coefficients for all metal ions, R^2^ were much closer to unity for graph t/q_t_ versus time. These results indicated that the pseudo-second order kinetic model satisfactorily fitted to describe the sorption of Pb^2+^, Cu^2+^, Ni^2+^ and Zn^2+^ onto CMSS/CA hydrogel and suggests that the chemisorption was the rate-controlling step. The chemical process involves the sharing or exchanging of the valence electron between sorbent and sorbate [16,43]. Therefore, the carboxylate groups played a significant role for the metal ions sorption through the ionic interaction between the metal ions and –COO^−^ groups of CMSS (Figure 8) in the present study.

#### 3.2.3. Effect of Initial Metal Ion Concentration

**Figure 10:**
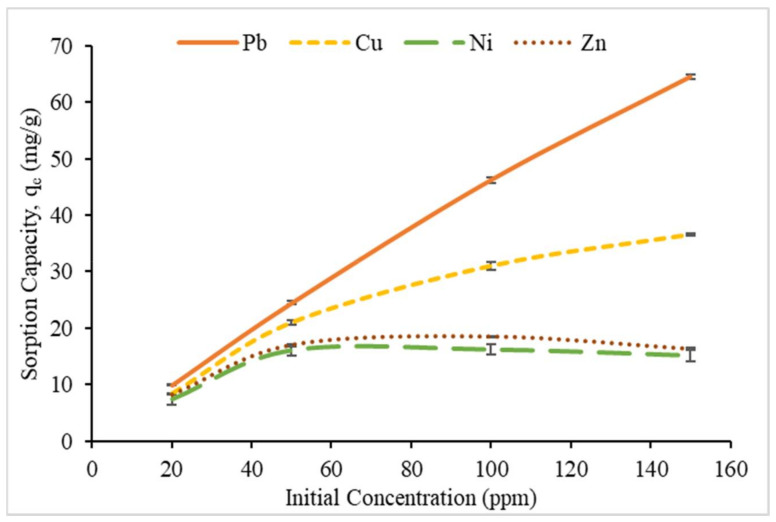
Effect of initial concentration on sorption capacity, q_e._

The effect of initial metal ion concentration on the sorption capacity was investigated using different initial metal ion concentrations (20, 50, 100 and 150 ppm) at room temperature with optimum pH and reaction time. Figure 10 illustrates the effect of initial concentration on the sorption capacity of CMSS/CA hydrogel. It clearly showed that the amount of sorbate (metal ions) per weight unit of sorbent increased with increasing the initial concentration of metal ion solution. Al-qudah et al. [43] and Zulfiqar et al. [44] claimed that initial concentration generates a concentration gradient that allows the driving force to overcome the resistance to mass transfer of metal ions between the aqueous and solid. Therefore, the higher the initial concentration of metal ion rises the driving force, the more attraction occurred between actives sites of sorbent and metal ions [45] increased the sorption capacity of CMSS/CA hydrogel. Also, the highest sorption for Pb^2+^ and Cu^2+^ occurred at 150 ppm metal concentration where sorption capacity values were 64.48 mg/g and 36.56 mg/g, respectively. However, the highest sorption for Ni^2+^ and Zn^2+^ was found at 100 ppm with a sorption capacity value of 16.21 mg/g, and 18.45 mg/g, respectively.

#### 3.2.4. Isotherm Study

Sorption isotherm is important to define the fraction of sorbate molecules that are divided between solid and liquid phases in equilibrium. In order to express the most appropriate correlation of equilibrium adsorption data, two isotherm models named Langmuir and Freundlich isotherms were used. The Langmuir isotherm assumes that monolayer coverage on the homogenous surface of the adsorbent and adsorbed molecules does not interact. Other than this, all active sites have an equal affinity to sorbate and constant sorption activation energy. The Freundlich isotherm was also employed to describe the adsorption capacity of adsorbate with non-uniform energy distribution over heterogeneous surfaces. This expresses reversible adsorption where the adsorbates are occupied in multilayer phases.

The linearized expression of isotherm equation is defined as follows [46]:

Langmuir isotherm

(6) $$\frac{C_{e}}{q_{e}}=\frac{1}{K_{L}q_{m}}+\frac{C_{e}}{q_{m}}$$

Freundlich isotherm

(7) $${\mathrm{lnq}}_{e}{=\mathrm{lnK}}_{F}+\frac{1}{n}{\mathrm{lnC}}_{e}$$

where C_e_ (mg/L) is the equilibrium concentration of metal ions, q_e_ (mg/g) is the amount of metal ions adsorbed at equilibrium, q_m_ (mg/g) is maximum sorption capacity, K_L_ (L/mg) represent Langmuir constant associated to the affinity of binding sites and adsorption energy, K_F_ (mg/g) is the Freundlich constant and n is the heterogeneity factor.

**Figure 11:**
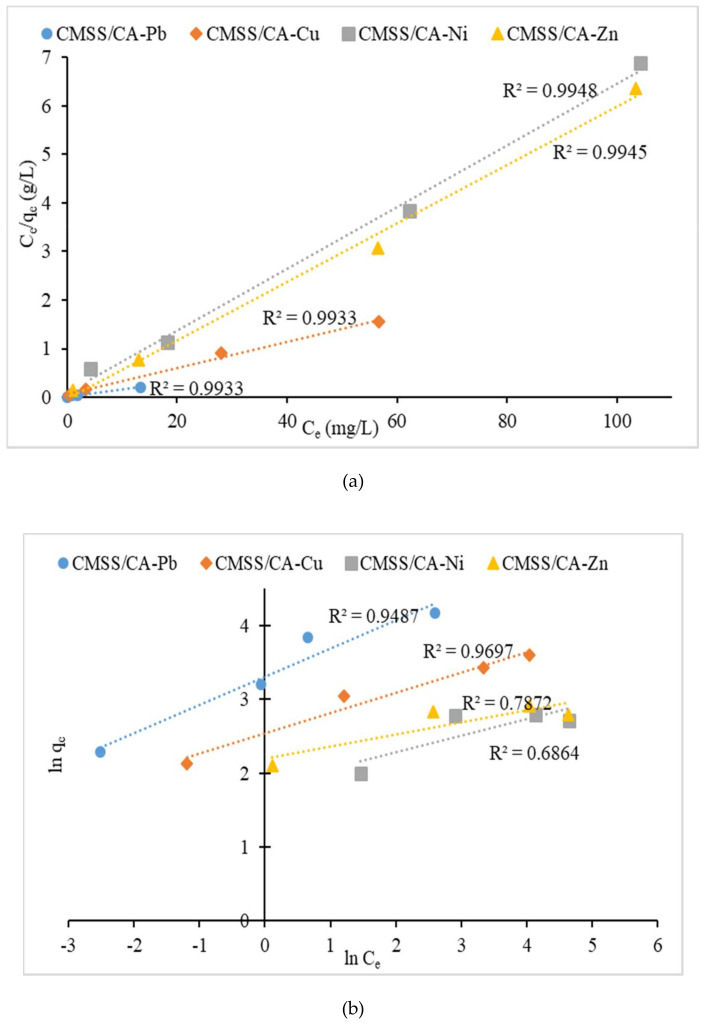
(**a**) Langmuir, and (**b**) Freundlich isotherm for sorption of metal ions onto CMSS/CA hydrogel.

The Langmuir isotherm graph of C_e_/q_e_ against C_e_ and Freundlich isotherm graph of ln q_e_ against ln C_e_ were plotted in Figure 11a,b, respectively. The linear Langmuir graph with the intercept of 1/K_L_q_m_ can be calculated to obtain Langmuir constant, K_L_ and the slope of 1/q_m_ provided theoretical maximum sorption. Besides that, the linear Freundlich graph gives information about heterogeneity factor, n from the slope of 1/n and Freundlich constant, K_F_ from the intercept ln K_F_. Table 2 indicates the isotherm parameter and it shows the experimental results of the sorption capacity of Pb^2+^, Cu^2+^, Ni^2+^ and Zn^2+^ were in good agreement with the Langmuir model. The correlation coefficient, R^2^ in Langmuir graphs nearly to value of 1, compared to R^2^ value in Freundlich graphs. Moreover, the maximum capacities (q_m_) acquired were close to experimental data of sorption at equilibrium, thus suggested that divalent metal ions sorption on the surface of CMSS/CA hydrogel was coated in monolayer coverage. The maximum capacity for Pb^2+^, Cu^2+^, Ni^2+^ and Zn^2+^ were 69.44 mg/g, 37.31 mg/g, 15.72 mg/g, and 16.58 mg/g, respectively. The K_L_ (L/mg) represented Langmuir constant associated with the affinity of binding sites. The results showed that the K_L_ value were arranged in the following order: Pb^2+^ > Ni^2+^ > Cu^2+^ > Zn^2+^. This indicated that the affinity of the CMSS/CA hydrogel binding sites towards Pb^2+^ was highest compared to other metal ions. Tran et al. [16] also showed a similar trend in Langmuir constant, K_L_ of the hydrogel as the binding energy of metal ions becomes stronger due to stronger electrostatic interaction between metal ion and sorbent.

#### 3.2.5. Effect of Temperature and Thermodynamic Study

**Figure 12:**
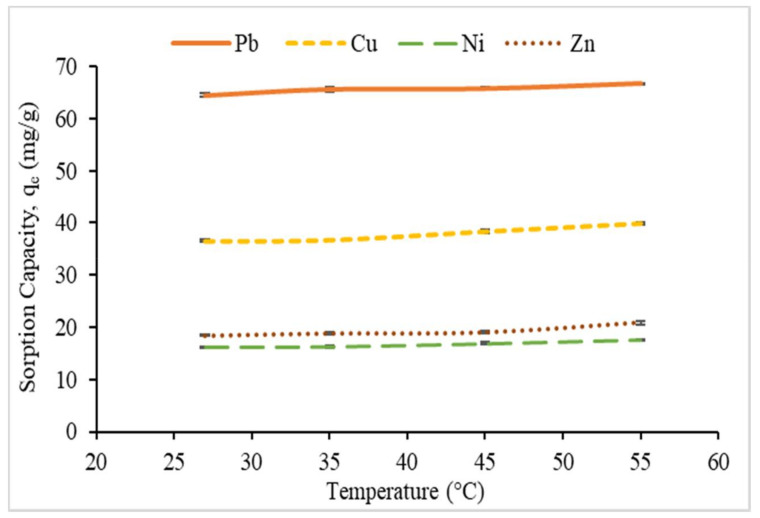
Effect of temperature on sorption capacity, q_e._

To study the thermodynamic parameter, the sorption procedure was performed in a water bath with various temperatures; 27 °C to 55 °C under optimum condition. The effect of reaction temperature on the sorption capacity is presented in Figure 12. Therefore, with the increase temperature from 27 to 55 °C, the sorption capacity Pb^2+^ onto CMSS/CA hydrogel increased from 36.56 mg/g to 39.86 mg/g. Also, other metal ions showed a similar trend in sorption capacity. Fosso-Kankeu et al. [47], as well reported similar findings and explained that, in order for metal ions to interact with sorption sites, sufficient energy is needed particularly for a large number of the metal ion. Increasing the temperature may reduce the viscosity of the aqueous solution and causes the greater diffusion rate of metal ions into the network of the hydrogel. Therefore, the sorption capacity of metal ions on CMSS/CA hydrogel increased. Despite the fact of higher temperatures resulted in higher sorption capacity, the percentage of increment in sorption capacity of Pb^2+^, Cu^2+^, Ni^2+^ and Zn^2+^ was insignificant with 3.41%, 9.03%, 8.27%, and 13.17%, respectively. In industrial applications, such a wastewater treatment, it is inapplicable to use high temperature since the operation would become unproductive and costly with high energy consumption. Therefore, it can be concluded that the optimum temperature in the sorption of heavy metal ions by CMSS/CA hydrogel was at room temperature.

**Figure 13:**
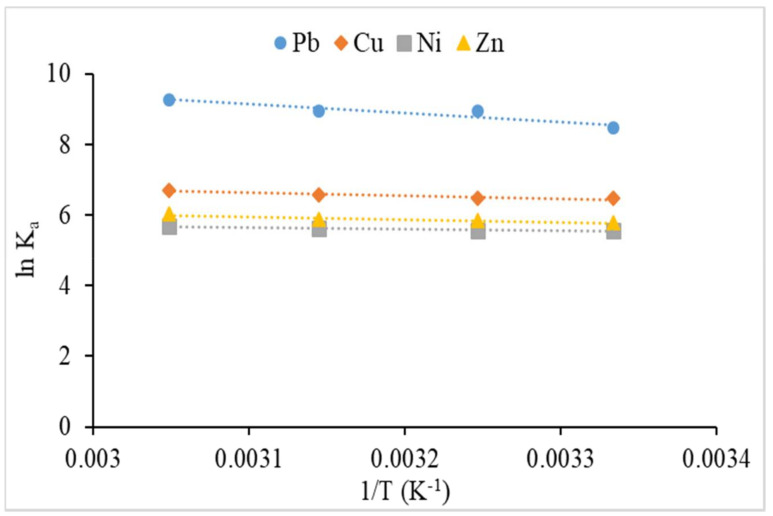
Van’t Hoff plot for thermodynamics parameter of metal ion sorption by CMSS/CA hydrogel.

The thermodynamic study of the sorption of heavy metals on sorbent was evaluated through parameters, such as standard change of Gibbs free energy (Δ*G°*, kJ/mol), entropy change (Δ*S°*, kJ/mol/K), and enthalpy change (Δ*H°*, kJ/mol). These thermodynamic parameters can be calculated according to the linear van’t Hoff equation [47],

(8) $${\mathrm{lnK}}_{a}=\;-\frac{\Delta G{}^{\circ}}{\mathrm{RT}}=\;-\frac{\Delta H{}^{\circ}}{\mathrm{RT}}+\frac{\Delta S{}^{\circ}}{R}$$

(9) $$K_{a}=\frac{q_{e}}{C_{e}}$$

where K_a_ (mL/g) is the adsorption equilibrium constant, q_e_ is the amount of metal ions adsorbed per unit mass of the adsorbent at equilibrium (mg/g), C_e_ (mg/L) is the concentration of metal ions at equilibrium, T (K) is the solution temperature and R is the ideal gas constant (8.314 × 10^−3^). The values of ΔH° and ΔS° can be calculated from the slope and intercept of graph ln K_a_ versus 1/T (Figure 13) while ΔG° was calculated using Equation (8). The thermodynamic parameters obtained are presented in Table 3. The enthalpies, ΔH° values for sorption of Pb^2+^, Cu^2+^, Ni^2+^ and Zn^2+^ on CMSS/CA hydrogel were positive values which suggested that the sorption process is an endothermic reaction. The positive value of entropy changes, ΔS° for all metal ions and indicated that the interface randomness increased between aqueous solution a swell as CMSS/CA hydrogel. In addition, the change in internal energy, ΔG° values for sorption of all metal ions was negative and showed that the reaction was spontaneous sorption under experimental condition. The more negative the value of ΔG° with increased temperature from 300–328 K, the more it revealed that higher temperature was favorable in the sorption of metal ions onto CMSS/CA hydrogel.

#### 3.2.6. Comparison of CMSS/CA Hydrogel with Polysaccharides-Based Hydrogel

Table 4 shows the tabulated comparison studies on the sorption capacity of various polysaccharides-based hydrogels as metal sorbent. By comparing the sorption capacity of CMSS/CA hydrogel with the maximum sorption capacity of the previous studies, the CMSS/CA hydrogel noticeably shows high sorption efficiency towards the Pb^2+^. Thus, the CMSS/CA hydrogel can be considered as a good alternative to be employed as bio-sorbent to remove heavy metal ions in wastewater treatment.

### 3.3. Selectivity Study

The selectivity of CMSS/CA hydrogel towards different metal ions (lead, copper, nickel and zinc) was studied under non-competitive and competitive conditions. Under the non-competitive condition, the metal ion sorption was carried out in a single metal ion solution while under a competitive solution it was done in a mixed metal ion solution. However, to examine the selectivity of CMSS/CA hydrogel, heavy metal removal was carried out with 0.05 g hydrogel in 25 mL of 20 ppm metal solution in pH 4 at room temperature for 30 min.

**Figure 14:**
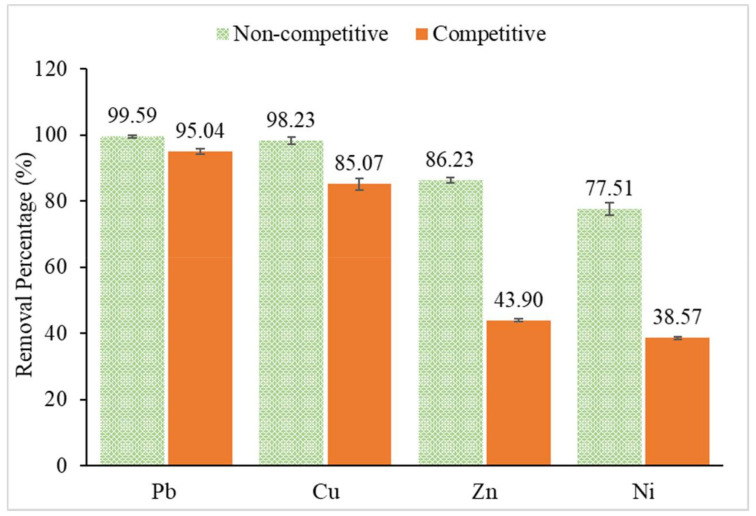
The removal percentage of Pb^2+^, Cu^2+^, Zn^2+^ and Ni^2+^ under non-competitive and competitive condition.

Figure 14 shows that in both non-competitive and competitive conditions, the CMSS/CA hydrogel was highly selective towards Pb^2+^. This is because Pb^2+^ ions have the highest atomic number, 82 and highest atomic radius compared to Cu^2+^, Ni^2+^ and Zn^2+^. Thus, the outermost electron of Pb^2+^ becomes further from the attractive force of the nucleus, making it more electropositive. The tendency of Pb^2+^ to lose its electron was higher to facilitate the interaction between the active sites of CMSS/CA hydrogel. Besides, a single metal ion solution showed better metal uptake than a mixture of metal ion solution. Furthermore, less competition occurred in single metal ion solutions due to the metal ions having the same atomic properties and reactivity, thus compete fairly towards binding sites in hydrogel [48]. While, in competitive conditions with multi heavy metal ions, the removal percentage of Pb^2+^ and Cu^2+^ was slightly decreasing in values, and Zn^2+^ and Ni^2+^ were reduced about half of the non-competitive removal percentage values.

Conversely, in both non-competitive and competitive conditions, the order of removal percentage of heavy metal ion: Pb^2+^>Cu^2+^>Zn^2+^>Ni^2+^. A similar trend was observed by Bhat et al. [49] in the removal of heavy metal ions from aqueous solution using crosslinked potato starch as adsorbent. This explained the sorption mechanism of Pb^2+^, Cu^2+^, Ni^2+^ and Zn^2+^ based on the coordination interaction of starch-metal complexes. In the case of Cu^2+^, Zn^2+^ and Ni^2+^, they are located side by side at period four of the periodic table and belong to the transition metal group. Despite these metal ions been divalent metal ion, the removal percentage of Cu^2+^, Ni^2+^ and Zn^2+^ were significantly different. This is attributed to different stability constants of formed chelates. As such, the removal percentage of Cu^2+^ was higher compared to Ni^2+^ and Zn^2+^ due to complexes that were formed between copper and the ligands which is most stable in accordance with Irving-Wiliam’s series. The Irving-Wiliams series refer to the stability of complexes formed by first-row transition metal ions which suggest the order: Mn^2+^ <Fe^2+^ < Co^2+^ < Ni^2+^ < Cu^2+^ > Zn^2+^ [50].

### 3.4. Desorption Study

The desorption study was done by using the Pb^2+^-loaded CMSS/CA hydrogel with a sorption capacity of 60.70 mg/g as a representative heavy metal ion to determine the capability of CMSS/CA hydrogel to desorb the Pb-loaded CMSS/CA hydrogel without the dissolution of the CMSS/CA hydrogel. In the present study, HCl was used as the desorption medium. When the CMSS/CA hydrogel loaded with Pb^2+^ and in contact with HCl, the bond between Pb^2+^ and active site of the carboxyl group in the hydrogel was disrupted [26]. Consequently, the metal ions leached out from the hydrogel and released into the desorption medium. From the result obtained, the desorption of Pb^2+^ from CMSS/CA hydrogel showed high desorption with the desorption percentage was 91.86%. This approves that the sorbed metal ions can be easily desorbed from CMSS/CA hydrogel. Hence, the Pb^2+^ solution could be recovered and concentrated to be used again. Moreover, it enables the disposal of used sorbent in a safer condition. Last, the desorption of heavy metal ion from CMSS/CA hydrogel is capable of avoiding secondary pollution in wastewater treatment.

## 4. Conclusions

The present study reveals that the CMSS/CA hydrogel successfully synthesized by chemically crosslinking with citric acid and can be employed as bio-sorbent to remove Pb^2+^, Cu^2+^, Ni^2+^ and Zn^2+^ from aqueous solution under several parameters. By characterizing the CMSS/CA hydrogel, the ester crosslinkages in hydrogel composition can be proved by the ester band in the FTIR spectrum. The CMSS/CA hydrogel has a porous structure captured in the SEM micrograph and has higher thermal stability compared to CMSS. Under optimum condition, the maximum capacity of Pb^2+^, Cu^2+^, Ni^2+^ and Zn^2+^ were 64.48, 36.56, 16.21 and 18.45 mg/g, respectively. The results of the kinetic study showed that the sorption of all heavy metal ions on CMSS/CA hydrogel obeyed the pseudo-second order kinetic and the rate was controlled by a chemical reaction, which includes the sharing or exchanging of the valence electron between the –COO^−^ group of CMSS/CA hydrogel and metal ions. According to the isotherm study, the sorption behavior of heavy metal ions on CMSS/CA hydrogel was well described by the Langmuir model. While, the sorption process of Pb^2+^, Cu^2+^, Ni^2+^ and Zn^2+^ on hydrogel was a spontaneous and exothermic reaction.

On the other hand, the selectivity study showed that Pb^2+^ was the most selective metal ion owing to a higher tendency to lose its electron and binds with active sites of the CMSS/CA hydrogel compared to other metal ions. Furthermore, the desorption of heavy metal ion was efficient in an acidic environment. Therefore, with high desorption percentage, the recovered heavy metal can be advantageous to be reused as secondary material in other industrial applications. From the sorption capacity of the CMSS/CA hydrogel towards Pb^2^, it shows that the CMSS/CA hydrogel is potentially applicable to be extended in industries, especially in wastewater treatment of industrial effluent for removal of Pb^2+^. As such, the column adsorption tests on the industrial effluent could be a study for further research.

## Figures and Tables

**Table 1:** Pseudo-first order and pseudo-second order sorption rate constant for sorption of metal ions by CMSS/CA.

Metal	q_e_,_exp_ (mg g^−1^)	Pseudo-First Order			Pseudo-Second Order		
		k_1_ (min^−1^)	q_e_,_calc_ (mg g^−1^)	R^2^	k_2_ (g mg^−1^ min^−1^)	q_e_,_calc_ (mg g^−1^)	R^2^
Pb	46.23	0.0387	7.737	0.9130	0.0109	47.17	0.9992
Cu	31.79	0.0465	3.396	0.9553	0.0254	32.26	1.0000
Ni	16.58	0.0138	1.108	0.2446	0.0616	16.47	0.9983
Zn	19.27	0.0302	2.146	0.9745	0.0283	19.61	0.9997

**Table 2:** Isotherm study for sorption of metal ions onto the CMSS/CA hydrogel.

Metal	q_e_,_exp_ (mg g^−1^)	Langmuir Isotherm			Freundlich Isotherm		
		K_L_ (L mg^−1^)	q_max_ (mg g^−1^)	R^2^	K_F_ (L^1/n^ mg^1/n−1^ g^−1^)	n	R^2^
Pb	64.48	0.9536	69.44	0.9933	27.34	2.625	0.9487
Cu	36.56	0.3851	37.31	0.9933	12.73	3.638	0.9697
Ni	16.21	0.6175	15.72	0.9948	6.303	4.505	0.6864
Zn	18.46	−1.317	16.58	0.9945	8.968	6.079	0.7872

**Table 3:** Thermodynamic parameters for sorption of metal ions by CMSS/CA hydrogel.

Metal	T (K)	1/T (K^−1^ × 10^−3^)	K_a_ (mL/g)	ln K_a_	ΔH (kJ mol^−1^)	ΔS (J mol^−1^ K^−1^)	ΔG (kJ mol^−1^)
Pb (II)	300	3.33	4833	8.483			−21.159
	308	3.25	7632	8.940	20.484	0.1395	−22.893
	318	3.15	7763	8.957			−23.681
	328	3.05	10627	9.271			−25.282
Cu (II)	300	3.33	645.6	6.470			−16.138
	308	3.25	654.9	6.485	6.793	0.07622	−16.605
	318	3.15	723.2	6.584			−17.406
	328	3.05	808.8	6.696			−18.259
Ni (II)	300	3.33	260.7	5.563			−13.876
	308	3.25	263.2	5.573	3.649	0.05830	−14.271
	318	3.15	276.4	5.622			−14.863
	328	3.05	294.7	5.686			−15.506
Zn (II)	300	3.33	326.5	5.788			−14.436
	308	3.25	346.7	5.848	6.284	0.06896	−14.975
	318	3.15	352.3	5.864			−15.504
	328	3.05	413.2	6.024			−16.427

**Table 4:** Comparison studies on the sorption of the heavy metal ion by various polysaccharides-based hydrogels as metal sorbent.

Sorbent	Heavy Metal Ion	Sorption Capacity (mg/g)	References
Gellan Gum/Graphene Oxide	Zn^2+^	120.48	[3]
Porous starch xanthate	Pb^2+^	107.36	[15]
Porous starch citrate	Pb^2+^	48.30	
Carboxymethyl Cellulose/sodium styrene	Pb^2+^	0.0036	[16]
sulfonate	Cu^2+^	0.0252	
	Ni^2+^	0.0267	
	Zn^2+^	0.0303	
Crosslinked carboxymethyl corn starch	Pb^2+^	76.53	[23]
Carboxymethyl Sago Starch-lactic acid	Pb^2+^	56.08	[28]
	Cu^2+^	17.19	
Crosslinked carboxymethyl konjac	Cu^2+^	25.50	[31]
glucomannan	Pb^2+^	29.20	
Oil palm bio-waste/Chitosan/multiwalled carbon nanotubes (MWCNTs)/Polyvinyl alcohol	Pb^2+^	30.03	[44]
CMSS/CA	Pb^2+^	64.48	Present study
	Cu^2+^	36.56	
	Ni^2+^	16.21	
	Zn^2+^	18.45	
